# Clinical Outcomes of Single Intramedullary K-Wire Fixation with Figure-of-Eight Suture Augmentation for Ulnar Styloid Base and Distal Ulnar Metaphyseal Fractures

**DOI:** 10.3390/jcm15197474

**Published:** 2026-09-25

**Authors:** Jaeheon Lee, Cheungsoo Ha, Jae Hoon Kang, Chanho Jeong, Kwangchoul Lee, Jae In Chung, Yeongsik Yun

**Affiliations:** 1 Department of Orthopedic Surgery, Saeson Hospital, Daejeon 34134, Republic of Korea; leejaeheon.kr@gmail.com (J.L.); hchs1123@gmail.com (C.H.); 2 Department of Medical Sciences, Graduate School of Ajou University, Suwon 16499, Republic of Korea; 3 Department of Plastic Surgery, Saeson Hospital, Daejeon 34134, Republic of Korea

**Keywords:** ulnar styloid fracture, distal radioulnar joint, Kirschner wire fixation, tension-band augmentation, distal radius fracture

## Abstract

**Background/Objectives**: The optimal fixation method for ulnar styloid base fractures remains controversial. Conventional dual K-wire fixation may be technically challenging in certain fracture configurations, whereas hook plate fixation provides rigid fixation but requires additional implant placement and surgical exposure. The purpose of this study was to evaluate the clinical and radiographic outcomes of single intramedullary K-wire fixation combined with figure-of-eight suture augmentation for ulnar styloid base and distal ulnar metaphyseal fractures. **Methods**: We retrospectively reviewed 60 patients who underwent fixation of ulnar styloid base or distal ulnar metaphyseal fractures associated with distal radius fractures between 2021 and 2024. The construct consisted of a single intramedullary K-wire augmented with a figure-of-eight Ethibond suture. Radiographic union, functional outcomes, pain, range of motion, and complications were assessed at final follow-up. **Results**: The median follow-up was 14 months (range, 12–60). Radiographic union was achieved in 58 of 60 patients (96.7%) at a mean of 9.6 ± 1.9 weeks. The median QuickDASH score was 4.5 (range, 0–18.2), the Modified Mayo Wrist Score was 91.7 ± 8.1, and the visual analogue scale score for ulnar-sided wrist pain was 1.7 ± 1.0. Two patients (3.3%) developed radiographic nonunion, but neither required revision surgery. No fixation failure, loss of reduction, deep infection, or revision fixation occurred. **Conclusions**: Single intramedullary K-wire fixation with figure-of-eight suture augmentation was associated with a high rate of radiographic union and favorable functional outcomes in ulnar styloid base and distal ulnar metaphyseal fractures. These findings support the clinical feasibility of this fixation construct.

## 1. Introduction

Distal ulna fractures involving the ulnar styloid base or distal metaphysis remain clinically important injuries because they are frequently encountered in association with distal radius fractures and may compromise the stabilizing anatomy of the distal radioulnar joint (DRUJ) [1,2]. Ulnar styloid fractures (USFs) accompany approximately 40–65% of distal radius fractures [3]. While fractures confined to the styloid tip are often managed nonoperatively, there is a general consensus that fractures involving the styloid base or extending into the distal metaphysis are more likely to require surgical fixation [1,2]. Their clinical significance extends beyond the osseous fragment itself; when the styloid base or foveal region is involved, the insertion of the triangular fibrocartilage complex (TFCC) may be disrupted, potentially jeopardizing DRUJ stability [4,5,6]. Furthermore, inadequately treated ulnar styloid base fractures are associated with a relatively high risk of nonunion, which may result in persistent ulnar-sided wrist pain and functional impairment [7].

Despite their clinical importance, the optimal management of these fractures remains controversial. Conservative treatment is appropriate for many stable ulnar styloid and distal ulna fractures, particularly when satisfactory reduction in the distal radius restores DRUJ stability [8,9]. However, displaced fractures involving the styloid base or distal metaphysis remain challenging. Consequently, increasing attention has been directed toward more aggressive surgical management strategies in recent years.

Among operative treatment options, dual K-wire fixation and hook plate fixation have emerged as the two most commonly utilized and frequently compared techniques [10,11]. Fixation using dual Kirschner wires remains attractive because it is familiar, technically straightforward, and minimally invasive. However, placement of two independent wires within a limited distal fragment may be technically challenging, particularly in certain fracture configurations. In such situations, maintaining stable reduction while avoiding fragment comminution can be technically demanding [11].

Hook plate fixation addresses some of these limitations by providing more rigid fixation and appears particularly useful in displaced or complex fracture patterns [12]. Nevertheless, hook plate techniques typically require a more extensive surgical exposure, with reported incisions extending 6–8 cm proximally from the ulnar styloid. In addition, implant prominence and soft-tissue irritation remain recognized complications because of the subcutaneous location of the distal ulna. The procedure may also place the dorsal sensory branch of the ulnar nerve at risk, potentially resulting in postoperative neurological symptoms [13]. Several authors have recently described lower-profile fixation strategies, including screw fixation augmented with tension-band constructs, absorbable implant augmentation, and arthroscopically assisted techniques [10,13,14]. However, a simple and reproducible fixation method using readily available implants has been less well characterized.

Therefore, the purpose of the present study was to evaluate the clinical and radiographic outcomes of distal ulna fractures involving the ulnar styloid base and/or distal metaphysis treated using a single intramedullary K-wire combined with figure-of-eight suture augmentation. We hypothesized that this technique would be associated with satisfactory radiographic and functional outcomes.

## 2. Materials and Methods

### 2.1. Study Design

This retrospective case series was conducted at a secondary referral institution specializing in hand and upper-extremity surgery in South Korea. The study was approved by the Institutional Review Board prior to data collection (IRB no. P01-202607-01-045). Medical records and radiographic images were retrospectively reviewed for all patients who underwent surgical fixation of distal ulna fracture involving the ulnar styloid base or distal metaphysis associated with distal radius fractures between January 2021 and December 2024.

### 2.2. Patient Selection

Patients were eligible for inclusion if they underwent operative treatment for a distal radius fracture and had a concomitant distal ulna fracture involving the ulnar styloid base or distal metaphysis requiring surgical fixation. Surgical fixation of the distal ulna was indicated when the fracture involved the ulnar styloid base or distal metaphysis and demonstrated one or more of the following characteristics: (1) displacement greater than 2 mm, (2) loss of cortical contact, or (3) persistent instability after distal radius fixation. Patients were excluded if they had isolated ulnar styloid tip fractures, severe open fractures, comminution requiring plate fixation, pathological fractures, previous wrist surgery, or insufficient follow-up data. A minimum follow-up period of 12 months was required for inclusion. The patient selection process is summarized in the STROBE flow diagram (Supplementary Figure S1). A total of 87 consecutive patients were screened, and 60 patients met the eligibility criteria and were included in the final analysis. Several patients treated during the earlier study years had extended follow-up into 2026, resulting in a right-skewed distribution of follow-up duration.

Fractures were categorized as either ulnar styloid base fractures or distal ulnar metaphyseal fractures based on preoperative radiographs and computed tomography (CT) scans. Distal ulnar metaphyseal fractures were defined as fractures in which the fracture line extended proximally beyond the ulnar head–neck junction.

### 2.3. Surgical Technique

All procedures were performed by two fellowship-trained hand surgeons (Y. Y., J. L.). Following fixation of the distal radius fracture, attention was directed to the distal ulna. A 4-cm longitudinal skin incision was made over the dorsal aspect of the ulnar styloid. Careful dissection was carried out to identify and protect the dorsal sensory branch of the ulnar nerve. The fracture site was exposed, and interposed soft tissue and hematoma were removed. Fracture reduction was achieved under direct visualization using a small reduction clamp or temporary Kirschner wire fixation when necessary.

After reduction, a single 1.0 or 1.2-mm Kirschner wire was inserted from the tip of the ulnar styloid in an intramedullary fashion across the fracture site into the proximal fragment. When the far cortex was reached, bicortical purchase was obtained. The K-wire served as the primary stabilizer, maintaining fracture alignment and resisting translational displacement. Reduction and wire position were confirmed fluoroscopically.

To augment fixation, a No. 0 Ethibond suture (Ethicon, Somerville, NJ, USA) was applied in a figure-of-eight configuration across the fracture site. The distal limb of the suture was passed beneath the peripheral rim of the TFCC while the proximal limb was secured through a transosseous tunnel created in the distal ulna. When creation of the transosseous tunnel with a needle alone was difficult because of dense cortical bone, a Kirschner wire was used to create a pilot hole. The suture was then tensioned and tied to generate compression across the fracture site and enhance rotational stability.

Following completion of the figure-of-eight construct, the distal end of the K-wire was cut, bent, and buried beneath the peripheral rim of the TFCC to minimize implant prominence and soft-tissue irritation (Figure 1). Final reduction and construct stability were confirmed fluoroscopically during passive wrist motion and forearm pronation-supination.

### 2.4. Postoperative Rehabilitation

A short-arm splint was applied for two weeks following surgery. Immediate active motion of the fingers was encouraged. After splint removal, gradual active wrist and forearm rotation exercises were initiated. Passive range-of-motion exercises were permitted from four weeks postoperatively. Strengthening exercises were permitted after radiographic evidence of fracture union. Return to unrestricted activities was allowed according to clinical and radiographic recovery.

#### Follow-Up and Implant Removal

Patients underwent clinical and radiographic follow-up at approximately 2-week intervals during the first 6 postoperative weeks and at approximately 4-week intervals thereafter until fracture union was confirmed. The ulnar K-wire was removed after 6 weeks in patients who developed symptomatic hardware irritation. In patients who elected to undergo distal radius implant removal after fracture union, the ulnar K-wire was generally removed during the same procedure. In patients with symptomatic K-wire irritation, earlier removal was performed after sufficient fracture stability had been achieved. Distal radius implants were typically removed after 6 months following fracture union. For K-wire removal, the previous incision was reopened, the capsule overlying the buried wire tip was partially incised, and the capsule was repaired after wire removal.

### 2.5. Outcome Assessment

Clinical outcomes were evaluated at final follow-up and included visual analogue scale (VAS) score for ulnar-sided wrist pain, Quick Disabilities of the Arm, Shoulder and Hand (QuickDASH) score, Modified Mayo Wrist Score (MMWS), wrist range of motion, and forearm pronation-supination [15,16]. QuickDASH and VAS were obtained as patient-reported outcome measures. MMWS and wrist and forearm range of motion were assessed by the treating hand surgeons during routine outpatient follow-up. Grip strength, as a component of the MMWS, was measured using a hand dynamometer during outpatient follow-up and recorded as a percentage of the contralateral unaffected side.

Radiographic assessment was performed by the treating hand surgeons using serial posteroanterior and lateral wrist radiographs obtained during routine postoperative follow-up. Fracture union was defined as bridging trabeculation across the fracture site accompanied by the absence of tenderness at the fracture site. Radiographic nonunion was defined as persistent absence of bridging trabeculation at ≥6 months after surgery. Time to union was defined as the interval from surgery to the first follow-up visit at which these criteria were fulfilled. Time to union, maintenance of reduction, and implant-related complications were recorded. Complications including neurologic symptoms, infection, hardware irritation, nonunion, and secondary surgical procedures were reviewed. For patients who underwent implant removal, final clinical outcomes were assessed after implant removal.

#### Statistical Analysis

Descriptive statistics were calculated using SPSS software (version 25, IBM Corp., Armonk, NY, USA). Continuous variables are presented as mean ± standard deviation, whereas follow-up duration and QuickDASH score are presented as median (range). Categorical variables are presented as frequencies and percentages.

## 3. Results

A total of 60 patients met the inclusion criteria and were included in the final analysis. The mean age at the time of surgery was 57.1 ± 12.8 years, and 41 patients (68.3%) were female. The dominant hand was involved in 33 patients (55.0%), and the right side was affected in 35 patients (58.3%). According to the AO/OTA classification, distal radius fractures were classified as type A in 7 patients (11.7%), type B in 11 patients (18.3%), and type C in 42 patients (70.0%). Volar locking plate fixation was used for distal radius fixation in 58 patients (96.7%), whereas alternative fixation methods were used in 2 patients (3.3%). Forty-seven patients (78.3%) had ulnar styloid base fractures, and 13 patients (21.7%) had distal ulnar metaphyseal fractures. The median follow-up duration was 14 months (range, 12–60 months) (Table 1).

Radiographic union was achieved in 58 of 60 patients, resulting in a union rate of 96.7%. The mean time to union was 9.6 ± 1.9 weeks (Figure 2). Two patients (3.3%) developed nonunion; however, no cases of fixation failure or loss of reduction were observed during the follow-up period (Figure 3).

Clinical outcomes at final follow-up were favorable. The median QuickDASH score was 4.5 (range, 0–18.2), the mean Modified Mayo Wrist Score was 91.7 ± 8.1, and the mean visual analogue scale score for ulnar-sided wrist pain was 1.7 ± 1.0. Wrist range of motion demonstrated satisfactory recovery, with a mean extension of 72.8° ± 9.6°, flexion of 74.5° ± 8.8°, ulnar deviation of 24.1° ± 5.3°, radial deviation of 18.4° ± 4.1°, pronation of 83.6° ± 6.2°, and supination of 84.1° ± 5.8°. For the grip-strength component of the MMWS, grip strength relative to the contralateral side was 90–100% in 27 patients (45.0%), 75–89% in 32 patients (53.3%), and 50–74% in 1 patient (1.7%); no patient had grip strength below 50%.

Hardware removal was performed in 46 patients (76.7%) at a mean of 8.4 ± 3.1 months after surgery. In most cases, the ulnar K-wire was removed concurrently with elective removal of the distal radius implant rather than as a separate procedure for ulnar-sided symptoms. Four patients (6.7%) underwent earlier K-wire removal because of symptomatic hardware irritation. The remaining 14 patients retained the ulnar K-wire during the available follow-up. No subsequent wire-related procedure was required in the 14 patients who retained the K-wire during available follow-up. Complications were generally mild and successfully managed without major secondary procedures. Transient neurologic symptoms related to irritation of the dorsal sensory branch of the ulnar nerve occurred in 3 patients (5.0%) and resolved with conservative treatment. One patient (1.7%) developed a superficial wound infection, which responded to oral antibiotics without surgical intervention. Hardware irritation was observed in 4 patients (6.7%) and was successfully treated with early removal of the ulnar K-wire after fracture stability had been achieved (Figure 4).

Two patients (3.3%) demonstrated persistent radiographic nonunion. However, both patients reported only mild symptoms and maintained satisfactory wrist function. Because neither patient demonstrated significant pain nor functional limitation, additional surgical procedures aimed at achieving bony union were not performed. No cases of implant failure, loss of reduction, deep infection, or revision fixation were observed during the follow-up period (Table 2).

## 4. Discussion

In this retrospective case series, single intramedullary K-wire fixation combined with figure-of-eight suture augmentation was associated with a radiographic union rate of 96.7% and favorable functional outcomes in patients with displaced ulnar styloid base and distal ulnar metaphyseal fractures. The principal finding of this study is therefore the clinical feasibility of this relatively simple fixation construct rather than evidence of superiority over established fixation techniques.

The optimal fixation method for ulnar styloid base fractures remains controversial [9]. Recently, direct comparative evidence between tension-band wiring and hook plate fixation demonstrated that both techniques can achieve satisfactory union and functional outcomes when appropriately selected according to fracture morphology [11]. Tension-band fixation appeared advantageous for smaller distal fragments because of its lower implant profile and less invasive nature, whereas hook plate fixation provided greater stability in larger fragments and fractures with metaphyseal extension. The present construct uses a different approach, combining a single intramedullary K-wire with nonabsorbable figure-of-eight suture augmentation. Although this configuration may reduce the amount of metallic fixation required, the present study was not designed to determine whether these technical characteristics translate into clinical advantages over tension-band wiring or hook plate fixation. A descriptive comparison with selected published comparative clinical studies is provided in Supplementary Table S1.

Previous authors have reported favorable outcomes using headless compression screw fixation, suture-anchor augmentation, transosseous suture fixation, absorbable fixation devices, and arthroscopically assisted TFCC-based repair techniques [14,17,18,19]. Although these methods have demonstrated encouraging clinical results, many require specialized implants, increased procedural complexity, or additional cost. Consequently, no single operative fixation strategy has been established as optimal for all displaced ulnar styloid base and distal ulna fracture patterns. The favorable outcomes observed in the present series suggest that stable fixation may be achieved using a simple construct composed of readily available implants and standard surgical instruments.

Several previous studies have questioned the necessity of routine fixation of ulnar styloid fractures associated with distal radius fractures. In particular, Souer et al. reported that unrepaired fractures of the ulnar styloid base did not adversely affect outcomes following plate fixation of distal radius fractures [9]. They found no significant difference in outcomes between fractures displaced less than 2 mm and those displaced 2 mm or more. Therefore, displacement alone may not be sufficient to distinguish patients who require fixation. In our present study, however, the operative indications also included loss of cortical contact or persistent instability after distal radius fixation, whereas Souer et al. [9] did not select patients on the basis of persistent DRUJ instability. Accordingly, the two cohorts differ in their selection criteria, although the present study cannot determine which specific ulnar styloid fractures require operative fixation.

The proposed mechanical rationale of the construct is straightforward. The intramedullary K-wire is intended to maintain fracture alignment and resist translational displacement, whereas the figure-of-eight suture is intended to provide supplementary compression and rotational stability. By passing the distal limb beneath the peripheral rim of the TFCC and securing the proximal limb through a transosseous tunnel, the construct follows the general principles of tension-band fixation. However, because no biomechanical testing was performed, these proposed mechanical properties remain theoretical.

Although radiographic nonunion occurred in two patients (3.3%), neither patient reported substantial pain or functional limitation, and neither required revision surgery. This observation is consistent with previous reports suggesting that radiographic nonunion of ulnar styloid fractures does not necessarily correlate with clinically significant disability [8,9]. Similarly, although hardware removal was performed in 76.7% of patients, this finding should be interpreted within the context of local practice patterns in South Korea, where elective implant removal following fracture union is commonly performed because of patient preference and healthcare accessibility [20]. In many cases, removal of the ulnar K-wire was performed concurrently with distal radius implant removal and therefore should not be regarded as a surrogate marker of implant-related complications. Notably, symptomatic hardware irritation occurred in only four patients (6.7%), all of whom experienced symptom resolution after implant removal.

Several limitations should be acknowledged. First, the retrospective design introduces the possibility of selection bias. Second, the study lacked a control group, precluding direct comparison with conventional dual K-wire fixation, tension-band wiring, or hook plate fixation. Accordingly, the present findings should be interpreted as evidence of clinical feasibility rather than superiority over established fixation methods. Third, clinical and radiographic outcomes were assessed during routine follow-up by the treating surgeons without independent or blinded evaluation, and no formal interobserver or intraobserver reliability analysis was performed for radiographic union. Fourth, standardized postoperative DRUJ stability testing and a DRUJ-specific patient-reported outcome measure were not available because of the retrospective study design. Therefore, the present study cannot determine whether this technique provides specific benefits with respect to DRUJ stability. Fifth, ulnar styloid base and distal ulnar metaphyseal fractures were analyzed as a single cohort, and the relatively small number of metaphyseal fractures precluded reliable fracture subtype-specific analysis. Sixth, the follow-up duration may not have been sufficient to evaluate long-term degenerative changes in the DRUJ. Finally, biomechanical testing was not performed, and the proposed mechanical advantages of the construct remain theoretical.

Despite these limitations, this study provides clinical data from a relatively large case series evaluating single intramedullary K-wire fixation combined with figure-of-eight suture augmentation for ulnar styloid base and distal metaphyseal fractures. The favorable clinical and radiographic outcomes support the clinical feasibility of this construct and provide a basis for future comparative and biomechanical studies.

## 5. Conclusions

Single intramedullary K-wire fixation with figure-of-eight suture augmentation was associated with a high rate of radiographic union and favorable functional outcomes in this retrospective series of ulnar styloid base and distal ulnar metaphyseal fractures. These findings support the clinical feasibility of this fixation construct.

## Figures and Tables

**Figure 1:**
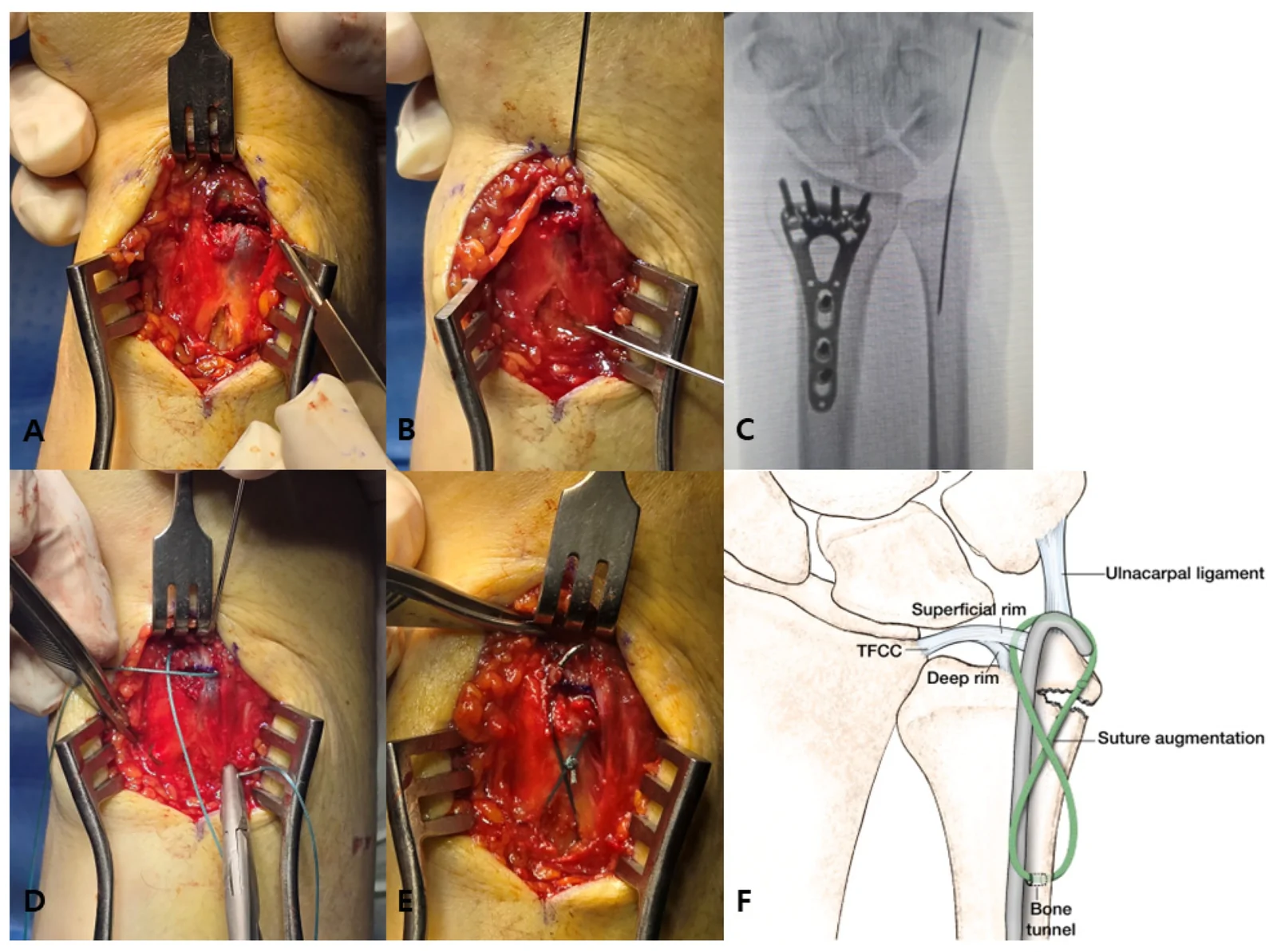
Surgical photographs and schematic illustration of single intramedullary K-wire fixation with figure-of-eight suture augmentation. (**A**) Exposure of the ulnar styloid base fracture through a 4-cm dorsal approach. (**B**) Fracture reduction and fixation with a single intramedullary K-wire. (**C**) Intraoperative fluoroscopic confirmation of fracture reduction and K-wire position. (**D**) Passage of the Ethibond suture beneath the peripheral rim of the triangular fibrocartilage complex. (**E**) Completion of the figure-of-eight suture augmentation through a transosseous tunnel in the distal ulna. (**F**) Schematic illustration demonstrating the final construct consisting of a single intramedullary K-wire and figure-of-eight suture augmentation.

**Figure 2:**
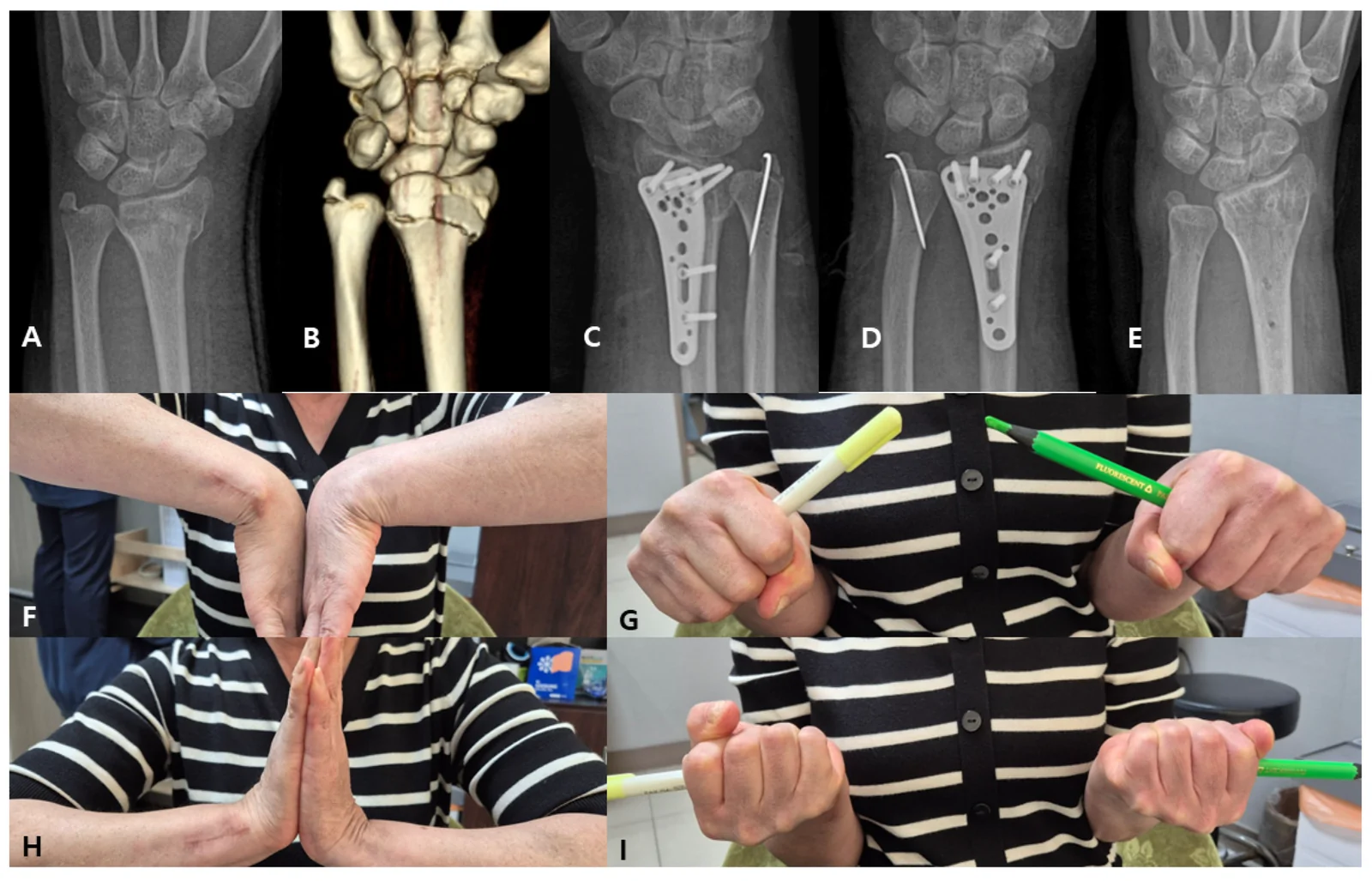
AO/OTA type C2 distal radius fracture associated with an ulnar styloid base fracture. (**A**,**B**) Preoperative radiograph and 3D computed tomography demonstrating the distal radius fracture and associated ulnar styloid base fracture. (**C**) Immediate postoperative radiograph following fixation of fractures. (**D**) Radiograph obtained at final follow-up demonstrating complete fracture union. (**E**) Radiograph obtained after implant removal. (**F**–**I**) Clinical photographs demonstrating near-full range of wrist motion, including flexion, extension, pronation, and supination.

**Figure 3:**
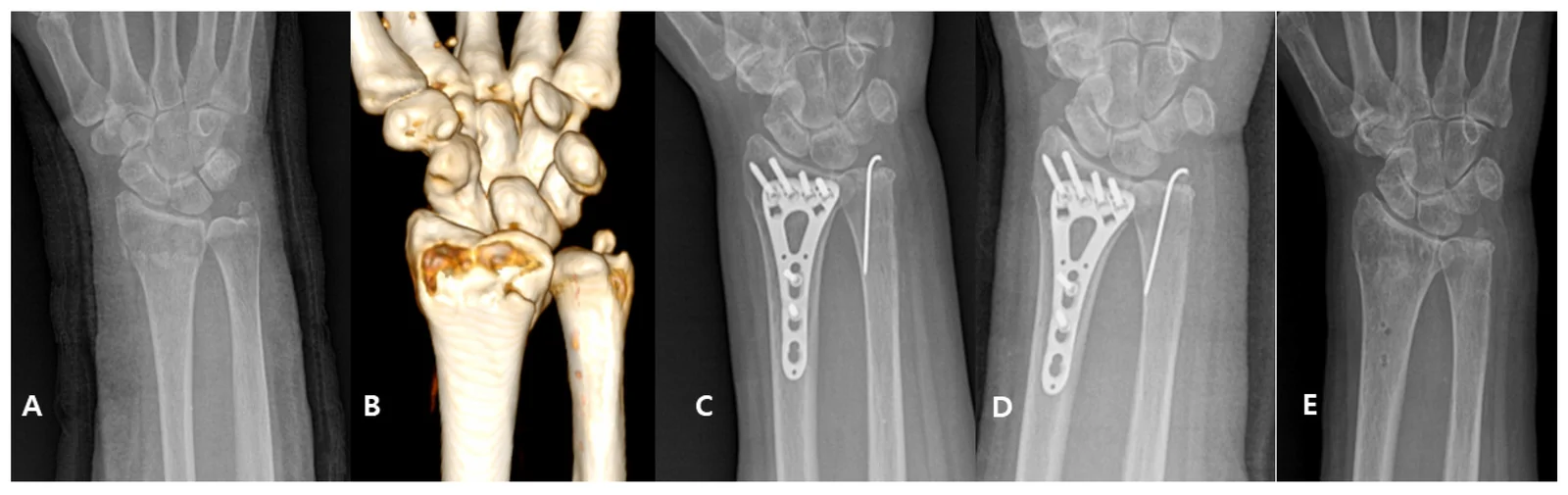
Representative case of radiographic nonunion with an AO/OTA type C2 distal radius fracture associated with an ulnar styloid base fracture. (**A**,**B**) Preoperative radiograph and 3D computed tomography demonstrating the distal radius fracture and associated ulnar styloid base fracture. (**C**) Immediate postoperative radiograph showing satisfactory fracture reduction and fixation. (**D**) Follow-up radiograph demonstrating persistent radiographic nonunion of the ulnar styloid fracture despite maintenance of alignment. (**E**) Radiograph obtained after implant removal. The patient remained minimally symptomatic with satisfactory wrist function and did not require revision surgery despite persistent radiographic nonunion.

**Figure 4:**
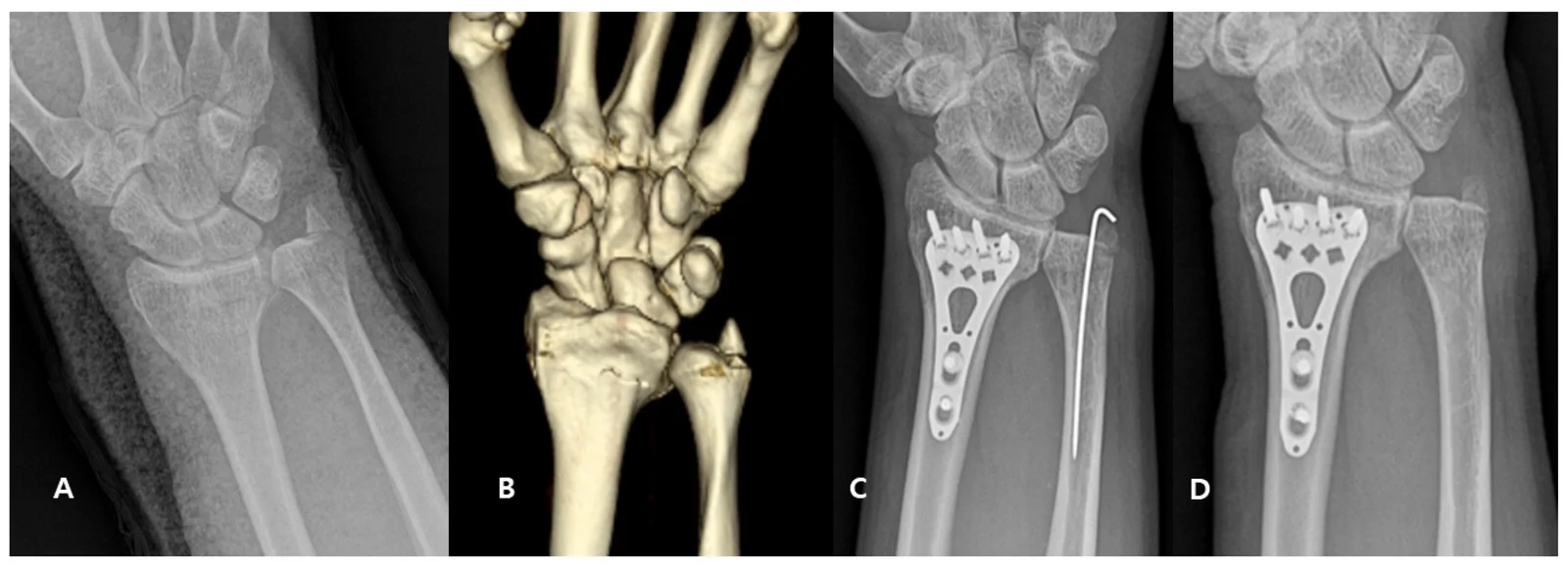
Representative case of early K-wire removal due to hardware irritation in a 60-year-old man with an AO/OTA type B2 distal radius fracture associated with an ulnar styloid base fracture. (**A**,**B**) Preoperative radiograph and 3D computed tomography demonstrating the distal radius fracture and associated ulnar styloid base fracture. (**C**) Immediate postoperative radiograph showing satisfactory fracture reduction and fixation. (**D**) Radiograph obtained 6 weeks postoperatively after early removal of the ulnar K-wire because of symptomatic hardware irritation, demonstrating maintained fracture reduction and radiographic union of the ulnar styloid fracture.

**Table 1:** Patient Demographics and Injury Characteristics.

Value	Variable
60	Number of patients
57.1 ± 12.8	Age (years), mean ± SD
19/41	Sex (Male/Female)
33 (55.0%)	Dominant hand involved, n (%)
	Sidedness
35 (58.3%)	• Right side, n (%)
25 (41.7%)	• Left side, n (%)
	AO/OTA classification of distal radius fracture
7 (11.7%)	• A type
11 (18.3%)	• B type
42 (70.0%)	• C type
	Distal radius fixation method
58 (96.7%)	• Volar locking plate
2 (3.3%)	• Other fixation methods
47 (78.3%)	Ulnar styloid base fracture, n (%)
13 (21.7%)	Distal ulnar metaphyseal fracture, n (%)
14 (12–60)	Follow-up duration (months), median (range)

Abbreviations: SD, standard deviation; AO/OTA, AO Foundation/Orthopaedic Trauma Association.

**Table 2:** Clinical and Radiographic Outcomes.

Result	Outcome
96.7% (58/60)	Union rate
9.6 ± 1.9	Time to union (weeks)
4.5 (0–18.2)	QuickDASH, median (range)
91.7 ± 8.1	MMWS
1.7 ± 1.0	VAS score for ulnar-sided wrist pain
	Wrist ROM
72.8 ± 9.6°	• Extension
74.5 ± 8.8°	• Flexion
24.1 ± 5.3°	• Ulnar deviation
18.4 ± 4.1°	• Radial deviation
83.6 ± 6.2°	• Pronation
84.1 ± 5.8°	• Supination
76.7% (46/60)	Hardware removal rate
8.4 ± 3.1	Time to hardware removal (months)
	Complication
5.0% (3/60)	• Transient neurologic symptoms
1.7% (1/60)	• Superficial infection
6.7% (4/60)	• Hardware irritation
3.3% (2/60)	• Nonunion

Abbreviations: QuickDASH, Quick Disabilities of the Arm, Shoulder and Hand; MMWS, Modified Mayo Wrist Score; VAS, visual analogue scale; ROM, range of motion.

## Data Availability

The data that support the findings of this study are available from the corresponding author upon reasonable request.

## Supplementary Materials

The following supporting information can be downloaded at https://www.mdpi.com/article/10.3390/jcm15197474/s1. Figure S1: STROBE flow diagram of this study; Table S1: Descriptive comparison of selected published comparative clinical studies of ulnar styloid fracture fixation.
