# Supplementary material for: Clinical Outcomes of Single Intramedullary K-Wire Fixation with Figure-of-Eight Suture Augmentation for Ulnar Styloid Base and Distal Ulnar Metaphyseal Fractures
Source: J Clin Med. 2026 Sep 25;15(19):7474. doi: 10.3390/jcm15197474 (PMC13666427; doi:10.3390/jcm15197474)
Supplement: Supplementary file 1 [file jcm-15-07474-s001.zip › jcm-4533611-supplementary.pdf]

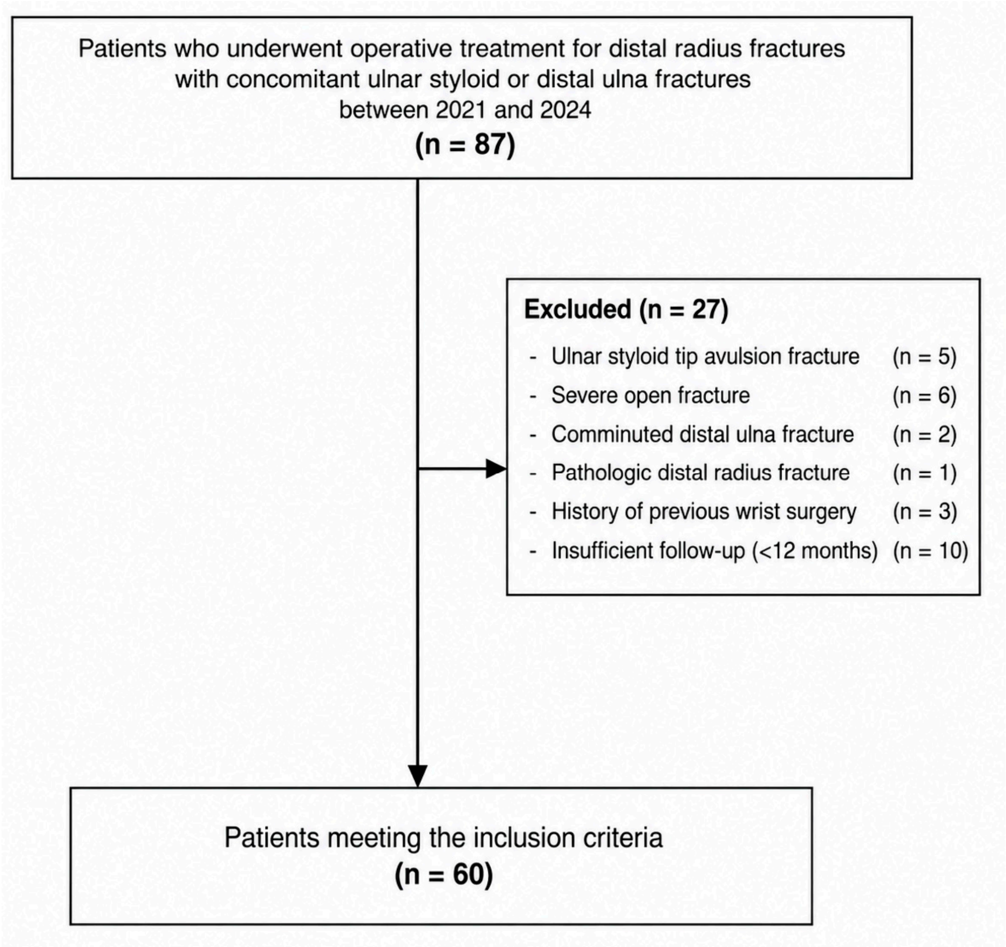

Figure S1. STROBE flow diagram of this study.

Supplementary Table S1. Descriptive comparison of selected published comparative clinical studies of ulnar styloid fracture fixation

Study	Design / cohort	Fixation technique(s)	Radiographic outcome	Functional and implant-related findings
Chen et al. (2020) [17]	Retrospective comparative study; n = 31; symptomatic USF with DRUJ instability; fixation selected according to fragment size/morphology; concomitant DRF in 9/31	Suture-anchor fixation + DRUJ pinning (n = 10) vs TBW (n = 21)	Union 94% overall (10/10 vs 19/21)	At 2-year follow-up, MMWS, QuickDASH, VAS, and bone-related outcomes did not differ significantly between groups. Implant-related complications were more frequent with TBW.
Kang et al. (2024) [11]	Retrospective comparative study; n = 109	TBW (n = 53) vs hook plate (n = 56)	Union 96% overall; time to union was shorter with hook plate for type 3–4 fractures	Flexion-extension was similar; pronation-supination favored TBW. Grip strength, DASH, and VAS did not differ significantly.
Acar et al. (2025) [10]	Retrospective comparative study; n = 71	K-wire (n = 19) vs TBW (n = 27) vs headless compression screw (n = 25)	Nonunion, DRUJ subluxation, and bone resorption were similar among groups	ROM, QuickDASH, and MMWS were similar. Implant irritation and the need for secondary surgery were more frequent with TBW. Grip strength was lower with K-wire fixation than with TBW
Present study	Retrospective case series; n = 60; concomitant DRF in 60/60	Single intramedullary K-wire + figure-of-eight nonabsorbable suture	Union 96.7% (58/60); mean time to union 9.6 ± 1.9 weeks	QuickDASH 4.5 (0–18.2); MMWS 91.7 ± 8.1; VAS 1.7 ± 1.0. Hardware irritation 6.7%

Note: Data are presented as reported in the original studies and cannot be interpreted as direct comparative evidence. Reference numbers correspond to the main manuscript. Abbreviations: USF, ulnar styloid fracture; DRUJ, distal radioulnar joint; TBW, tension-band wiring; MMWS, Modified Mayo Wrist Score; QuickDASH, Quick Disabilities of the Arm, Shoulder and Hand; DASH, Disabilities of the Arm, Shoulder and Hand; VAS, visual analogue scale; ROM, range of motion.
